# Injurious Effects of a Rubber Plate

**Published:** 1884-07

**Authors:** 


### CORRESPONDENCE.

### Injurious Effects of a Rubber Plate.

Mrs. W——, a widow, aged forty-five, came to us complaining of her plate hurting her mouth. The plate of red vulcanite had been worn twelve years with but little more than the usual amount of annoyance, until recently. On examination, the mouth presented a fearful condition. Wherever the plate came in contact with the membrane the latter was very much inflamed and thickened, appearing something like a piece of raw beefsteak. The whole anterior portion of the ridge was soft and flabby. There was so much absorption that the plate had cut a groove for itself, the outer wall of which hung down over the edge of the plate like a lip. This flap was especially prominent on the left side from the median line to the locality of the first bicuspids, and about a third of an inch wide. On the right side was something of the same appearance, but not so far advanced. It was the worst case of absorption by vulcanite we ever saw.

An impression was taken, as we wished to keep a model, and to prevent the further wearing of the plate that was retained, an astringent mouth wash was given. After this had been used about two weeks, the membrane was much improved. A second impression was taken, models and dies made, and an aluminum plate fitted, which was worn about ten days without the teeth, as the patient was too poor to afford a gold plate or so much resetting. Then another model was made, the same plate being used, and the teeth attached with rubber. The plate was made to come out over and cover the flap before mentioned. There had been so much absorption of the ridge in front, that, in order to bring out the lips sufficiently, it was necessary to build out with wax a great deal, so that the rubber was fully one-fourth of an inch thick between the plate and teeth.

We saw the patient a few days ago, and found that after eighteen months the membrane is entirely normal in appearance, of a pale pink color, and firm. Of course the ridge in front is still soft like cartilage, as it doubtless is. There was no need of a second swaging of the plate, as we expected at first; due, probably, to the precaution taken before making the plate. The aluminum plate is but little discolored, and shows no signs of disintegration. K. C. M.

